# Prognostic Significance of DR-70 Levels in Dysplastic Colorectal Polyps

**DOI:** 10.1155/2013/275392

**Published:** 2013-11-21

**Authors:** Atakan Yesil, Gul Babacan Abanonu, Yasar Colak, Nurcan Paker, Can Gonen

**Affiliations:** ^1^Department of Gastroenterology, Haydarpaşa Numune Training and Research Hospital, Istanbul, Turkey; ^2^Department of Internal Medicine, Haydarpaşa Numune Training and Research Hospital, Istanbul, Turkey; ^3^Department of Gastroenterology, Medeniyet University, Istanbul, Turkey; ^4^Duzen Laboratories, Istanbul, Turkey

## Abstract

*Background*. To investigate the relationship between DR-70 serum levels and dysplastic colon polyps. *Materials and Methods*. A total of 130 patients with adenomatous polyps detected by colonoscopy and divided into two groups including low versus high grade polyp, along with 50 healthy blood donors were included in the study. Blood samples from each participant were analyzed for serum CEA and DR-70 levels. *Results*. No statistically significant differences were observed between the two groups in terms of age or gender. The median DR-70 level was 0.5 **μ**g/mL in the healthy control group and 1.1 **μ**g/mL in group 1b (i.e., the high grade polyp) (*P* < 0.001). DR-70 was higher in group 1b as compared to group 1a (*P* < 0.001). However, the median DR-70 values for the low grade polyp group (i.e., group 1a) and the control group were similar (*P* = 0.067). In order to determine independent predictors of high grade dysplasia, CEA, DR-70, polyp size, and age parameters were subjected to multiple logistical regression analyses via the Enter method; the model was statistically significant (*P* < 0.001). *Conclusions*. DR-70, a marker used to measure FDP, which is generated by all major cancers, is a potential marker to identify patients with advanced adenomatous polyps, that is, precursors of colorectal cancer.

## 1. Introduction

Excluding skin cancers, colorectal cancer (CRC) is the third most commonly diagnosed cancer after breast and lung cancer and the second leading cause of death in both sexes [1]. Strong evidence indicates that screening programs can reduce the incidence and mortality of CRC; indeed, early diagnosis is very important for long-term survival with this type of cancer [2]. In order to promote long-term survival, various screening programs have been developed to detect early stage CRC [3]. Among these screening programs, the most preferred method is the colonoscopy, but it is not an ideal screening method because it is expensive and uncomfortable and involves the risk of several complications including bowel perforation and hemorrhaging [4]. Despite extensive research, serum/fecal biomarkers that offer high sensitivity and specificity for the detection of early stage CRC have not yet been identified [4, 5].

Previous studies have reported that there is a strong relationship between increased fibrin/fibrinogen degradation product (FDP) levels and the presence of cancer [6–9]. According to these studies, tests for FDPs may be useful for the prediction of patient survival. Indeed, FDPs are tumor markers in many types of cancer and FDP concentrations are correlated to the presence, stage, progression, and prognosis of CRC [10]. 

The DR-70 ELISA is the first *in vitro* diagnostic test to have been approved for colon cancer screening by the USFDA since 1982 when carcinoembryonic antigen (CEA) was approved [10]. In contrast to tests that measure a single source of FDP, the DR-70 ELISA assay measures FDP generated from all major cancer-induced FDP production pathways [11, 12].

In this study, we investigated the relationship between serum DR-70 levels and the presence of dysplastic polyps, that is, premalignant lesions in the adenoma-carcinoma sequence of CRC. We assessed the ability of DR-70 to predict the presence of premalignant lesions before they become intramucosal adenocarcinomas, that is, an early stage of colon cancer.

## 2. Patients and Methods

This study included 130 patients with adenomatous polyps as detected by colonoscopies at the Gastroenterology Clinic of the Haydarpaşa Numune Training and Research Hospital and 50 healthy blood donors who composed the control group following confirmation of lack of any colon polyps on colonoscopy. Subjects were excluded from the study if they had CRC, impaired hepatic or renal function, diabetes mellitus, an uncontrolled infection, familial adenomatous polyposis, or conditions that could affect fibrinolysis and/or thrombosis (e.g., deep vein thrombosis, pulmonary embolism, or anticoagulant use). Accordingly, patients with false positive results for DR-70 including 3 patients with Crohn's disease, 2 patients with autoimmune hepatitis, 2 patients with infection, and in the sera of 4 patients with incompletely clotted blood during serum preparation were excluded from the present analysis. 

The demographic and clinical characteristics of the subjects, including age, gender, and colonoscopy findings at the time of diagnosis, were recorded via a questionnaire form. The study protocol was approved by the hospital's ethics committee and all study subjects provided written informed consent.

Carcinomas restricted to the epithelial layer without invasion into the lamina propria were classified as noninvasive high grade neoplasia.

A 5 mL sample of peripheral blood was obtained from each subject (i.e., both the patients with the adenomatous polyps and the healthy control subjects). The serum CEA and DR-70 levels and the hemogram for each participant were analyzed in the biochemistry laboratory of Haydarpaşa Numune Training and Research Hospital. The serum DR-70 concentration was measured following the manufacturer's protocol for the AMDL-ELISA DR-70 kit (AMDL Inc., Tustin, CA, USA).

### 2.1. Statistical Analysis

The Statistical Package for Social Sciences (SPSS) for Windows, v. 16.0, was used for all statistical analyses. The normal distribution of the continuous variables was analyzed using the one-sample Kolmogorov-Smirnov test. In addition to descriptive statistical methods, a one-way analysis of variance (ANOVA) was used to compare normally distributed parameters in more than two independent groups, whereas the Kruskal-Wallis test was used for parameters that did not show a normal distribution. If the results of the one-way ANOVA were significant, pairwise post hoc tests were performed using Tukey's honestly significant difference (HSD) test. For parameters that did not show a normal distribution, the Mann-Whitney *U* test was performed to test the significance of the pairwise differences, and the results were evaluated using the Bonferroni correction. Depending on the distribution of the data, Student's *t*-test or the Mann-Whitney *U* test was used to compare qualitative data from two independent groups. The chi-square test or Fisher's exact test was used for intergroup comparisons of quantitative data. The sensitivities and specificities of the immunoassay were calculated at various threshold concentrations. Thus, the best cutoff values for the DR-70 and CEA immunoassay were obtained via the receiver operating characteristics (ROC) curve analysis using the MedCalc statistical software 12.2.1 (MedCalc Inc., Mariakerke, Belgium) with 95% confidence intervals. Correlations between findings were assessed using Pearson's or Spearman's correlation analysis depending on the distribution of the data. When significant differences were observed between groups for certain parameters, these parameters were entered into a multiple logistic regression analysis to determine the independent predictors of high grade dysplasia. The odds ratios (OR) and 95% confidence intervals (CI) were determined with the accepted level for statistical significance set at *P* < 0.05.

## 3. Results

### 3.1. Demographics and Clinical Characteristics

We evaluated 130 patients (i.e., 72 males and 58 females) with a mean age of 60.08 ± 12.42 years (i.e., range of 26 to 89 years) in whom adenomatous polyps had been detected via colonoscopy and 50 healthy blood donors (i.e., 22 males and 28 females) with a mean age 58.0 (6.2) years (i.e., range of 49 to 72 years) who served as a control group. No statistically significant differences were observed between the groups in terms of age (*P* = 0.14) or gender (*P* = 0.18).

Among the patient group, the median number of individual polyps found in the colonoscopy was five, and the median polyp size was 5.5 mm. The polyps were most abundant in the rectum and least abundant in the cecum. Fecal occult blood was detected in 25% of the patients. The median DR-70 concentration was 0.80 *μ*g/mL, and the mean CEA concentration was 2.18 ng/mL.  Table 1 shows the demographic and clinical characteristics of the patient group. The subjects in the patient group were subdivided into two groups according to the dysplastic grade of the polyp including low grade dysplasia (*n* = 72, 37 males and 35 females; mean age of 60.1 (SD: 13.2; range 26–87) years) and high grade dysplasia (*n* = 58, 35 males and 23 females; mean age of 60.1 (SD: 11.5, range 28–89) years) groups. The two groups were similar in terms of age (*P* = 0.99) and gender distribution (*P* = 0.31).

When the control, low grade, and high grade groups were compared, significant differences were observed in the CEA levels (*P* < 0.001), serum DR-70 (*P* < 0.001) concentrations, and the polyp size (*P* = 0.02) (Table 1). Compared to the control group, the mean CEA concentration was significantly higher in patients with low grade (*P* < 0.001) and high grade (*P* < 0.001) polyps, whereas no significant difference was observed between the two patient subgroups (*P* = 0.72). The median DR-70 concentration in patients with high grade polyps was significantly higher than that of the control group (i.e., 1.1 *μ*g/mL versus 0.5 *μ*g/mL, resp., *P* < 0.001) and that of the patients with low grade polyps (*P* < 0.001). On the other hand, the median DR-70 concentration for patients with low grade polyps was similar to that of the control group (*P* = 0.07) (Table 1).

### 3.2. Correlation between CEA, DR-70, and Clinical Parameters

A significant positive correlation was observed between the CEA and DR-70 concentrations (*r* = 0.23; *P* = 0.002) and DR-70 concentration showed significant positive correlation with polyp size (*r* = 0.501; *P* < 0.001) (Table 2).

### 3.3. Multiple Logistic Regression Analysis for Factors Predicting High Grade Dysplastic Polyps

To determine the independent factors that can predict high grade dysplastic polyps, the blood CEA levels, the blood DR-70 levels, the polyp sizes, and the ages of the participants were entered into a multiple logistic regression analysis (Table 3). This model was statistically significant (*P* < 0.001) with a Nagelkerke *R* ^2^ value of 0.599. The DR-70 concentration (*P* < 0.001) was found to be independent predictor of high grade polyps. Each unit increase in the DR-70 concentration increased the likelihood of having a high grade dysplastic polyp 104.799-fold (95.0% CI 16.47–666.74).

### 3.4. ROC Curves for CEA and DR-70 in Differentiation of Dysplasia from the Healthy Control

**Figure 1:**
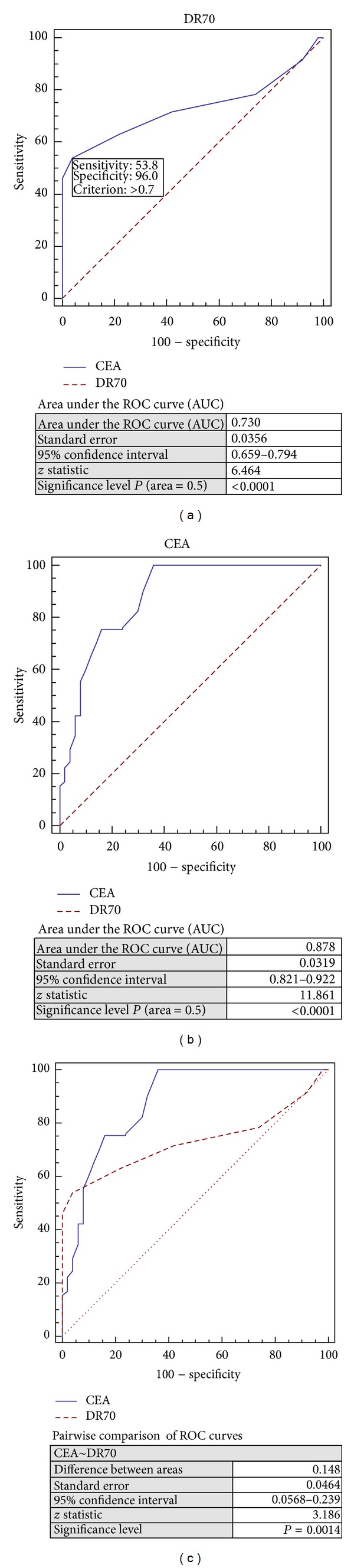
Receiver operating characteristic (ROC) curve for the diagnostic ability of (a) DR-70, (b) CEA, and (c) DR-70 versus CEA to differentiate the presence of dysplasia from the healthy control.

When the receiver operating characteristic (ROC) curve was drawn to investigate the diagnostic ability of DR-70 and CEA to distinguish the presence of dysplasia from the healthy control group, the most suitable cutoff value for DR-70 was 0.7 *μ*g/mL with sensitivity and specificity of 53.9% (95% CI 44.9–62.6) and 96.0% (95% CI 86.3–99.5), respectively. The most suitable cutoff value for CEA was 1.14 ng/mL with sensitivity and specificity of 100.0% (95% CI 97.2–100.0) and 64.0% (95% CI 49.2–77.1), respectively. ROC curves for CEA and DR-70 significantly differed in terms of differentiating dysplasia from the healthy control group (*P* = 0.0014). The area under the curve (AUC) was 0.730 (95% CI 0.659–0.794, *P* < 0.0001) for DR-70 and 0.878 (95% CI 0.821–0.922, *P* < 0.0001) for CEA (Figures 1(a)–1(c)).

### 3.5. ROC Curves for CEA and DR-70 in Differentiation of Low Grade versus High Grade Dysplasia

**Figure 2:**
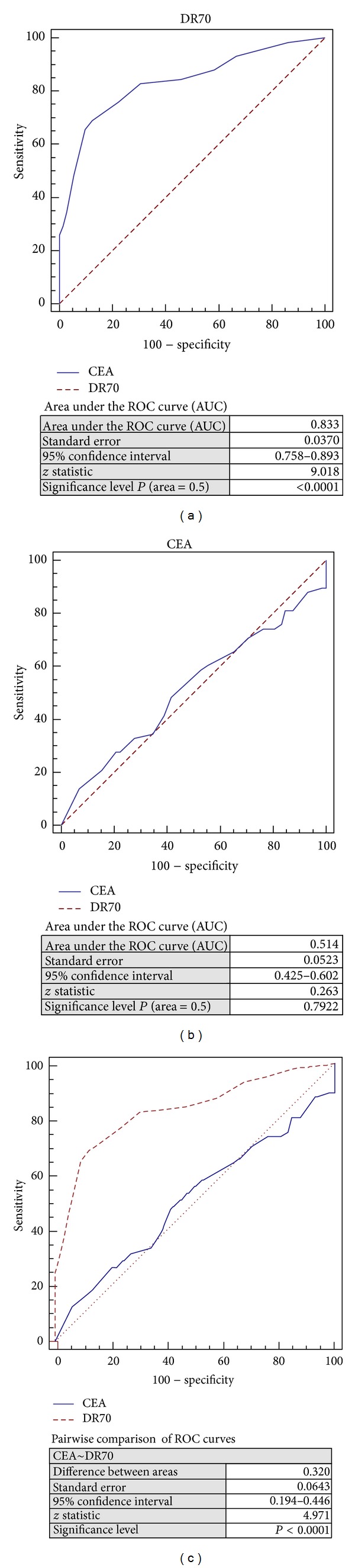
Receiver operating characteristic (ROC) curve for the diagnostic ability of (a) DR-70, (b) CEA, and (c) DR-70 versus CEA to differentiate low grade dysplasia from high grade dysplasia.

When the receiver operating characteristic (ROC) curve was drawn to investigate the diagnostic ability of DR-70 and CEA in differentiation of low grade versus high grade dysplasia, the most suitable cutoff value for DR-70 was 0.9 *μ*g/mL with sensitivity and specificity of 69.0% (95% CI 55.5–80.5) and 87.5% (95% CI 77.6–94.1), respectively. The most suitable cutoff value for CEA was 3.5 ng/mL with sensitivity and specificity of 89.7% (95% CI 78.8–96.1) and 0.0% (95% CI 0.0–0.5), respectively. ROC curves for CEA and DR-70 significantly differed in terms of differentiation of low grade versus high grade dysplasia (*P* < 0.0001). The area under the curve (AUC) was 0.833 (95% CI 0.758–0.893, *P* < 0.0001) for DR-70 and 0.514 (95% CI 0.425–0.602, *P* = 0.7922) for CEA (Figures 2(a)–2(c)).

## 4. Discussion

Intramucosal adenocarcinomas do not invade beyond the mucosa or muscularis mucosae. Any cancerous invasion beyond the muscularis mucosae to the submucosal area is considered invasive [13]. Aberrant crypt foci formation, that is, an intermediate step between normal colonic epithelium and adenoma development, is considered the earliest morphological precursor of epithelial neoplasia. This is followed by the formation of a small adenoma [14]. Further growth leads to dysplastic changes and finally to the development of invasive cancer. This model is called the adenoma-carcinoma sequence [15]. Dysplasia in adenomas is classified as low, medium, or high grade (i.e., carcinoma *in situ*) based on cytological and architectural features. Intramucosal adenocarcinomas can be distinguished from high grade dysplasia by the presence of lamina propria invasion [16]. Adenomas with high grade dysplasia are considered “advanced” lesions. Another criterion of advanced lesions is defined as a size that is larger than 1 cm with a villous structure. Herein, we analyzed potential predictive markers with 130 patients with adenomatous polyps and 50 healthy individuals as a control group [17, 18].

The 5-year survival rate of patients with early stage colorectal carcinomas is around 90%. Many screening programs have been developed in order to detect these tumors as early as possible [19]. The first tumor marker that was approved by the USFDA is CEA, which was first identified in 1965; currently, CEA is the most commonly used serum biomarker of CRC [20]. Although originally thought to have high sensitivity and specificity for CRC, CEA is more sensitive in patients with advanced disease than those with early stage disease; indeed, less than 40% sensitivity has been observed in patients with early stage CRC (i.e., A and B rated cancers according to Dukes classification), that is, the very population, that is, the target of screening programs [21]. Therefore, although CEA is useful in the followup of these patients, the use of CEA as a screening test is not recommended [22]. DR-70 ELISA is a new *in vitro* diagnostic test for CRC that measures the serum concentrations of fibrin and FDPs [23]. Cancer increases FDP concentrations via coagulation and fibrinolysis. Both u-PA and tissue factor (TF) affect the production of FDP and have been reported to increase in cancer patients. The enzyme u-PA transforms inactive plasminogen into active plasmin, whereas TF activates the coagulation cascade by activating thrombin, which then converts fibrinogen into fibrin. The plasmin degradation products of fibrinogen and fibrin are different. The plasmin degradation of fibrinogen produces X and Y fragments as intermediate products and D and E fragments as end products. In contrast, the plasmin degradation of fibrin produces D-dimers as the end product. The DR-70 test measures the products of both pathways, whereas other tests measure only a single FDP. Previous research has established that FDPs are valuable tumor markers in many types of cancer, including colorectal, lung, and ovarian cancers, and FDP concentrations correlate to the presence, stage, progression, and prognosis of cancer [24].

In a study in Germany of 85 patients with gastrointestinal cancers and 100 healthy individuals, the serum DR-70 concentration was found to be significantly higher in patients with advanced stage cancer as compared to that of those with early stage cancer. Since only 30 of these patients had CRC, this marker could not be shown to be organ-specific. In the current study, we excluded patients who had developed true CRC because our primary objective was to evaluate the ability of this marker to screen for early stage tumors [25].

Evaluation of DR-70 concentrations in various tumor types has shown that its highest sensitivity is in patients with stomach cancer (i.e., 92.6%) and the lowest sensitivity is in patients with rectal cancer (i.e., 66.7%). Serum DR-70 concentrations were significantly higher in patients with advanced adenomas as compared to healthy individuals without any polyps. Polyp location was not associated with DR-70 concentration [26]. Future studies with sample sizes may help elucidate any differences in DR-70 concentrations that may depend on tumor localization.

In a study in the USA in which 75 serum samples were obtained from 39 healthy controls and 36 patients with recurrent CRC, the researchers found that the combined use of DR-70 and CEA had a sensitivity of 75% and a specificity of 58.97%. The optimal cutoff points for DR-70 and CEA were 3 *μ*g/mL and 5 ng/mL, respectively, which are higher than the median DR-70 and CEA concentrations in patients with adenomas in the present study (i.e., 0.8 *μ*g/mL and 2.1 ng/mL, resp.). These differences may be caused by the differences in the study groups; more specifically, CRC patients were included in the earlier study, whereas only adenoma patients were included in the current study [26].

This study does have several limitations. For example, we did not include CRC patients in the sample population. Moreover, we could not include serrated adenomas because only five of our patients presented with this condition. Lastly, the patient sample size, that is, 130 cases, may not be sufficiently large to determine the cutoff level between dysplastic lesions and healthy controls.

Indeed, higher levels of DR-70 were also reported in patients with malignancies of the lung, nasopharynx, tongue, gastrointestinal tract, breast, ovary, and prostate gland [10, 27] and the likelihood of false positive results in nonmalignant gastrointestinal diseases such as lung infections and bronchitis was emphasized [28]. In this regard it seems notable to indicate that, given the identification of false positive results for DR-70 in the presence of infection, autoimmune diseases, inflammatory events, and fibrinolysis in our study population, none of these patients were included in the control or patients group subjected to the present analysis.

Cancers are characterized by irregularities at the cellular level. As these tumors progress, irregularities occur at the system level including the coagulation system. In cancer patients, the coagulation system can be affected by the inappropriate activation of either or both the coagulation and/or fibrinolytic pathways. Dysregulation of the coagulation system has major negative effects on cancer patients because of the involvement of this system in homeostasis and immunity [27]. We know that DR-70 is a marker that measures FDP, which is generated by all types of cancer, especially from stimulating both the coagulation and fibrinolytic systems [28].

## 5. Conclusion

To our knowledge, this study is the first to evaluate DR-70 as a marker in patients with advanced adenomatous polyps, that is, precursors of CRC. The diagnosis of early stage CRC using serum markers without the need for invasive interventions, like colonoscopies, will offer simpler and more cost-effective approaches. Further, prospective randomized studies involving more patients are needed to determine whether DR-70 ELISA can serve as a marker for early stage CRC.

## Figures and Tables

**Table 1:** Comparisons of demographic characteristics and clinical parameters in the control, low grade dysplasia, and high grade dysplasia groups.

	Controls (*n* = 50)	Patients			
		Low grade dysplasia (*n* = 72)	High grade dysplasia (*n* = 58)	Total (*n* = 130)	*P* value
	Mean (SD)				
Age (years)	58.0 (6.2)	60.1 (13.2)	60.1 (11.5)	60.1 (12.4)	0.53^a^

Gender	*n* (%)				
Male	22 (44.0)	37 (51.4)	35 (60.3)	72 (55.4)	0.23^b^
Female	28 (56.0)	35 (58.6)	23 (39.7)	58 (44.6)	
FOB positivity	—	18 (25)	13 (22.4)	31 (23.8)	0.73

	Median (min–max)				
Number of polyps	—	5 (1–6)	5 (1–6)	5 (1–6)	0.50^c^
Polyp size (mm)	—	5 (0–20)	6.0 (0–60)	5.5 (0–60)	0.02^c^
DR-70 (*μ*g/mL)	0.5 (0.2–0.8)	0.6 (0.3–1.4)	1.1 (0.3–4.3)	0.8 (0.3–4.3)	<0.001^d^

	Mean (SD)				
CEA (ng/mL)	1.0 (0.7)	2.1 (0.7)	2.2 (1.0)	2.2 (0.8)	<0.001^a^

^a^One-way ANOVA, ^b^Chi square test, ^c^Mann-Whitney *U*, ^d^Kruskal-Wallis test.

**Table 2:** Correlation between CEA, DR-70, and clinical parameters.

	CEA (ng/mL)		DR-70 (*μ*g/mL)	
	*r*	*p*	*r*	*p*
Polyp location	−0.008^a^	0.93	0.108^a^	0.22
Polyp size	−0.029^a^	0.74	0.501^a^	<0.001
DR-70 (*μ*g/mL)	0.230^a^	0.002	—	—
CEA (ng/mL)	—	—	0.230^a^	0.002
Age	0.026^b^	0.73	0.098^a^	0.19
Number of polyps	0.148^a^	0.09	−0.058^a^	0.51

*r*: correlation coefficient, ^a^Spearman's rho test, ^b^Pearson's test.

**Table 3:** Factors predicting high grade dysplastic polyps. Results of multiple logistic regression analysis.

	*P* value	OR	%95 CI	
			Lower bound	Upper bound
CEA	0.891	0.948	0.446	2.015
DR-70	**0.000**	104.816	16.476	666.832
Polyp size	0.069	0.897	0.797	1.009
Age	0.629	0.990	0.950	1.031

OR: odds ratio, CI: confidence interval.
